# Editorial: Proceedings of the 9th international symposium on the biology of vertebrate sex determination 2023

**DOI:** 10.3389/fcell.2024.1530367

**Published:** 2024-12-05

**Authors:** Talia Hatkevich, Dagmar Wilhelm

**Affiliations:** ^1^ Department of Cell Biology, Duke University Medical Center, Durham, NC, United States; ^2^ Department of Anatomy and Physiology, The University of Melbourne, Parkville, VIC, Australia

**Keywords:** sex determination, gonad, gonadal development, Müllerian duct, *Sry*

The survival of sexually reproducing species is reliant on proper formation of mature gametes and their subsequent fertilization, and in many organisms, this is dependent upon gonadal sex determination. Gonadal sex determination is the processes in which the bipotential genital ridge differentiates into an ovary or a testis, which can be driven by cues from genetic factors and/or environmental signals (Nagahama et al., 2021). In most mammals, sex determination is genetically dictated, initiated by the expression of the testis-promoting gene *Sry* from the Y chromosome (Koopman et al., 1991; Sinclair et al., 1990). Expression of *Sry* triggers the differentiation of the testes by activating *Sox9* in supporting precursor cells (Sekido and Lovell-Badge, 2008). This initiates a cascade of events, including the formation of Sertoli cells and Leydig cells, which are essential for producing sex hormones that subsequently drive male reproductive organ development (Svingen and Koopman, 2013). In the absence of the Y chromosome, the -KTS splice form of the transcription factor WT1 initiates ovarian development (Gregoire et al., 2023), which is associated with activated canonical WNT/β-catenin signalling and expression of the transcription factor FOXL2 (Chassot et al., 2008; Garcia-Ortiz et al., 2009; Gustin et al., 2016; Maatouk et al., 2008; Yao et al., 2004). Furthermore, to ensure proper gonad development, the testicular and ovarian program suppress each other (Kim et al., 2006). However, the mechanisms surrounding the nuanced processes of sex determination and sex-specific structures throughout vertebrates remain poorly defined. This Special Research Topic, “*Proceedings of the 9th International Symposium on the Biology of Vertebrate Sex Determination 2023*,” brings together articles that explore the complex mechanisms underlying sex determination and differentiation in vertebrates. The contributing pieces address key outstanding questions in the field, presenting novel findings and ideology that shed light on genetic, epigenetic, and hormonal regulation of gonadal development and sexual differentiation.

A central question within the field focuses on how genetic and epigenetic mechanisms orchestrate the sex-specific development of the bipotential gonad. Here, Ming et al. introduce a new testicular target gene of SOX9, *Trpc3*. This study shows that *Trpc3* is highly expressed in Sertoli cells during early gonadal development, and in *Sox9* knockout mice, *Trpc3* is downregulated. Inhibiting TRPC3 leads to reduced germ cell proliferation and endothelial cell apoptosis. Collectively, this work suggests that TRPC3 may mediate SOX9’s function in the testis, highlighting the role of *Trpc3* in gonadal development and its potential implications for understanding male infertility.

Expanding on the molecular landscape of sex determination, Stevant et al. explore the role of transposable elements (TEs), mobile genetic elements that can influence gene expression (Percharde et al., 2018). Sophisticated bioinformatics analysis identified TEs as key players in the regulation of sex-specific genes. Further, this study shows that TEs not only regulate gene expression through the production of TE-derived RNAs but also function as cis-regulatory elements that control the expression of sex-specific genes. TEs appear to play a crucial role in gonadal sex determination and differentiation, making TEs integral to the genetic program of sexual differentiation in vertebrates.

Sex determination mechanisms are diverse and can vary significantly across species. While mammals rely heavily on genetic factors, other vertebrates like zebrafish exhibit more flexible sex determination systems (Nagahama et al., 2021). In this research topic, Wilson et al. studied a wild strain of *Danio rerio*, which exhibits a ZZ/ZW chromosomal system. Using single cell sequencing, this work found that the presence of a W chromosome or fewer than two Z chromosomes is crucial for initiating ovarian development. Conversely, gonads with two Z chromosomes develop into testes, bypassing the juvenile ovary stage altogether. This discovery in zebrafish helps expand our understanding of the evolutionary forces that shape sex determination mechanisms across vertebrates.

The development of the Müllerian ducts, which give rise to the female reproductive tract, has long been a subject of study in sexual differentiation. In mammals, the ducts differentiate into the Fallopian tubes, uterus, and upper vagina, while in birds, the ducts form the oviducts. The role of anti-Müllerian hormone (AMH) in the regression of Müllerian ducts in males is well-established (Behringer, 1994; Behringer et al., 1990; Josso, Cate, et al., 1993; Josso, Lamarre, et al., 1993; Josso and Picard, 1986); however, there are remaining questions regarding species that exhibit sexual asymmetry, including chickens (Bakst, 1998). In female chickens, only the left Müllerian duct forms an oviduct. Tan et al. present a literature review on avian Müllerian duct asymmetry and proposes that local interactions between AMH and sex steroids could explain this phenomenon. Furthermore, while Müllerian ducts give rise to oviducts, the Wolffian ducts are precursors of the male reproductive tract. These reproductive tracts export gametes for subsequent fertilization. However, some species, like cyclostomes and basal teleost, lack genital ducts and instead possess genital pores to export gametes (Goodrich, 1930). These differences in gamete-exporting organs across vertebrates are discussed in a comprehensive review by Kanamori and Kobayashi. This review posits outstanding questions on the structure and development of gamete-exporting organs and emphasizes the importance of additional studies on cyclostomes, cartilaginous fishes, basal ray-finned fishes and teleost.

Testicular descent, the movement of testes from near the kidneys to the scrotum, is a key feature of most mammals, believed to be linked to the evolution of endothermy (Werdelin and Nilsonne, 1999). However, certain groups of mammals, particularly within *Afrotheria* and monotremes, exhibit either partial descent or internal testes (Sharman, 1970). Here, Menzies et al. explore the conservation and mechanism of marsupial testicular decent. Using phylogeny and gene analysis of hormone insulin-like peptide 3 (*Insl3*), the authors argue for a therian origin of INSL3 mediated testicular descent in mammals.

A critical aspect of sexual differentiation is the action of steroid hormones, which regulate gonadal function and fertility (De Gendt et al., 2004; Liu et al., 2009; Publicover and Barratt, 2011). Hormones like androgens, estrogens, progesterone, cortisol, and aldosterone influence testicular function through specific receptors, and disruption of these hormonal signals can have profound effects on fertility and sexual development. Matsuyama and DeFalco highlight the complex network of steroid hormones and their receptor function and localization. This review underscores the interplay of these signaling pathways and aims to serve as a resource for further investigation into hormonal mechanisms regulating of male reproductive health.

In addition to the gonad, the brain itself undergoes sexual differentiation, often influenced by steroid hormones released from the gonads (Arnold, 2009; Phoenix et al., 1959). However, genetic factors may also play a role in brain sex differentiation, independent of gonadal influence. Paylar et al. show that in rat brains sex-specific gene expression occurs prior to the onset of gonadal hormone action. The genes *Sry2*, *Eif2s3y*, and *Ddx3y* were found to be expressed at higher levels in males, perhaps contributing to the development of the male brain. These findings suggest that sex-specific genetic programs may contribute to brain differentiation alongside hormonal signals.

In conclusion, the field of vertebrate sex determination is rapidly evolving, with new findings shedding light on the genetic, epigenetic, and hormonal regulation of sexual differentiation. From the identification of novel testicular target genes to the exploration of TEs and homology of sex organs across species, this Special Research Topic highlights the complexity and diversity of sex determination mechanisms.
